# Loganin Inhibits Angiotensin II–Induced Cardiac Hypertrophy Through the JAK2/STAT3 and NF-κB Signaling Pathways

**DOI:** 10.3389/fphar.2021.678886

**Published:** 2021-06-14

**Authors:** Jia-jia Xu, Run-jing Li, Zheng-hao Zhang, Cui Yang, Shi-xiao Liu, Yan-ling Li, Min-wei Chen, Wei-wei Wang, Gong-ye Zhang, Gang Song, Zheng-rong Huang

**Affiliations:** ^1^ Department of Cardiology, The First Affiliated Hospital of Xiamen University, Xiamen, China; ^2^ Cancer Research Center, School of Medicine, Xiamen University, Xiamen, China

**Keywords:** loganin, angiotensin II, cardiac hypertrophy, cardiac fibrosis, inflammation, JAK2/STAT3, NF-kB

## Abstract

Loganin is an iridoid glycoside extracted from *Cornus officinalis*, which is a traditional oriental medicine, and many biological properties of loganin have been reported. Nevertheless, it is not clear whether loganin has therapeutic effect on cardiovascular diseases. Hence, the aim of the present study was to investigate the effect of loganin on Ang II–induced cardiac hypertrophy. In the present study, we reported for the first time that loganin inhibits Ang II–provoked cardiac hypertrophy and cardiac damages in H9C2 cells and in mice*.* Furthermore, loganin can achieve cardioprotective effects through attenuating cardiac fibrosis, decreasing pro-inflammatory cytokine secretion, and suppressing the phosphorylation of critical proteins such as JAK2, STAT3, p65, and IκBα. Besides, the outstanding findings of the present study were to prove that loganin has no significant toxicity or side effects on normal cells and organs. Based on these results, we conclude that loganin mitigates Ang II–induced cardiac hypertrophy at least partially through inhibiting the JAK2/STAT3 and NF-κB signaling pathways. Accordingly, the natural product, loganin, might be a novel effective agent for the treatment of cardiac hypertrophy and heart failure.

## Introduction

Cardiac hypertrophy is an early feature of many cardiovascular diseases and plays a pivotal role in heart failure and sudden death (Messerli et al., 2017). Cardiac hypertrophy, driven by pathological stimulation or biomechanical overload, is characterized by the elevated mass and size of cardiomyocytes. Originally, cardiac hypertrophy is an essential compensatory mechanism to maintain normal heart function; sustained hypertrophic stimulation will cause pathological cardiac hypertrophy, which is accompanied by increased protein synthesis, enhanced reactive oxygen species (ROS) production, activation of fetal cardiac genes, arrhythmia, inflammation, and cardiac fibrosis (Molkentin et al., 1998; Burchfield et al., 2013; Schiattarella and Hill, 2015). Angiotensin II (Ang II), a pivotal ingredient of the renin–angiotensin system (RAS), plays an important role in triggering hypertension and myocardial hypertrophy. According to currently published research, Ang II infusion induces myocardial hypertrophy *in vivo* and *in vitro*, which is mainly mediated through angiotensin II type 1 receptor (AT1R). It is extensively known that activation of AT1R, stimulated by Ang II treatment, is featured by the increased release of catecholamine, triggered vasoconstriction and vascular hypertrophy, induced hypertensive effect, and caused pathological cardiac remodeling (Hernandez-Hernandez et al., 2002; Lee et al., 2020). Additionally, in terms of the molecular mechanisms through which activation of AT1R evokes cardiac hypertrophy, numerous signaling pathways are well known to regulate this process. For instance, JAK2/STAT3 and nuclear factor-κB (NF-κB) signaling pathways are closely involved in the development of cardiac hypertrophy (Crowley, 2014; Tham et al., 2015). Although copious studies have reported the pathophysiological mechanisms of myocardial hypertrophy in mammals, the effective treatment approach is still lacking for cardiac hypertrophy at present. Therefore, identifying new treatment approaches is crucial for cardiac hypertrophy therapy, which will provide novel effective therapeutic strategies to reverse heart failure fundamentally.

Loganin (Log) is a unique component of the iridoid glycosides extracted from *Cornus officinalis*, which is a traditional Chinese medicine (Zhao et al., 2016). To date, increasing studies have shown that loganin has widespread biological activities, including anti-inflammation, anti-oxidation, anti-shock, anti-amnesia, anti-osteoporosis, anti-diabetes, hepatoprotection, neuroprotection, promotion of peripheral nerve repair, sedative and hypnotic actions, inhibition of diabetic nephropathy, and low side effects (Lee et al., 2009; Park et al., 2011; Jiang et al., 2012; Kim et al., 2015; Tseng et al., 2016; Chao et al., 2017; Mo et al., 2019; Shi et al., 2019; Yang et al., 2019; Cheng et al., 2020; Hu et al., 2020). For example, a study by Yu *et al.* proved that loganin has novel neuroprotective properties. In their study, it was found that loganin dramatically relieved neurite injury and oxidative stress through elevation of neurotrophic signaling and suppression of the RhoA/ROCK pathway (Tseng et al., 2019). In addition, the effects of loganin on hepatoprotection and nephroprotection have been reported in a type 2 diabetic mouse model. The study demonstrated that loganin possesses protective effects against hepatic and renal damage and other diabetic complications *via* inhibiting oxidation stress and advanced glycation end product formation (Yamabe et al., 2010). Of note, many reports have confirmed that loganin could suppress inflammatory responses by repressing the secretion of inflammatory cytokines and downregulating the activation of JAK2/STAT3 and NF-κB signaling pathways (Cui et al., 2018; Liu et al., 2020; Wang et al., 2020; Yuan et al., 2020). However, the therapeutic effects and potential mechanisms of loganin on cardiac hypertrophy are still unknown. Accordingly, the aim of the present study was to design experimental models of cardiac hypertrophy *in vivo* and *in vitro* and to investigate the effect of loganin on cardiac hypertrophy induced by Ang II.

## Materials and Methods

### Cell Culture

H9C2 rat embryonic cardiomyocytes were purchased from Procell Life Science & Technology Company (Wuhan, China). RLE-6TN rat alveolar type II epithelial cells were obtained from the cell bank of School of Medicine, Xiamen University (Xiamen, China). The H9C2 cells were cultured with high-glucose Dulbecco’s modified Eagle’s medium (DMEM). The RLE-6TN cells were maintained in DMEM/F-12. The media were supplemented with 10% fetal bovine serum (FBS), penicillin (100 U/mL), and streptomycin (100 μg/ml). Both of the cell lines were grown in a humidified incubator with 5% CO_2_ at 37°C. Based on previous literature reports and our preliminary test, we induced cardiomyocyte hypertrophy of H9C2 by applying angiotensin II (Ang II) (soluble in saline, cat.no. A9525, Sigma-Aldrich, United States) at 800 nM.

### Cell Viability Assay

Cell viability was assessed by MTT assay. H9C2 and RLE-6TN cells were dispensed in 96-well plates at 1x10^4^ cells per well. After 24 h of incubation, the cells were treated with loganin (Chengdu Ruifensi Biotech Co. Ltd., China, purity >98%) at serial concentrations (0, 6.25, 12.5, 25, 50, 100, 200, and 500 μM) for 24 h, and then, 20 μL of MTT solution (5 mg/ml) was added to each well and incubated at 37°C for 4 h. The medium was discarded carefully. Then, 150 μL of DMSO was added to each well. The absorbance at 490 nm was measured with a microplate reader to evaluate cell viability.

### Animals and Treatments

Six-week male C57BL/6 mice, weighing approximately 20–25 g, were purchased from Beijing Vital River Laboratory Animal Technology Co. Ltd., China. Mice were kept in an SPF environment, maintained at constant temperature and humidity, and given sufficient water and feed. Animal experiments were approved by the Ethics Committee of Xiamen University Medical School. ALZET^®^ Osmotic Pumps (2004 model, United States) were immersed in saline for 7 h in advance, and Ang II was dissolved in normal saline and injected into osmotic pumps. 1 cm transverse incision was made in the middle of the back in the mice, and the osmotic pump was implanted under the skin. The infusion rate of Ang II was 0.25 μL/h, and the concentration was 1.5 μg/kg per min. Meanwhile, the experiment was randomly divided into six groups with six mice in each group, which were the sham group, Ang II group, 50 mg/kg loganin group, 100 mg/kg loganin group, 200 mg/kg loganin group, and positive control group of 10 mg/kg telmisartan (cat.no. A8531, APExBIO Technology, Houston, Texas, United States). Loganin was dissolved in normal saline, and telmisartan was dissolved in 0.5% CMC-Na (sodium carboxylmethyl cellulose) solution. The mice were given 200 μL loganin or telmisartan in an intragastric gavage manner once every 2 days. The sham and Ang II groups received an equivalent volume of solvent. Tissue sampling was studied 30 days later.

### Echocardiography Measurement

Mice were anesthetized with 1.5% isoflurane. Then, their anterior chest hair was removed, and the ultrasound coupling agent was evenly smeared. Echocardiographic parameters were obtained by a VisualSonics high-resolution Vevo 2100 system (VisualSonics, Toronto, Canada).

### Blood Pressure Measurement

The blood pressure was detected by the tail-cuff method. The intelligent non-invasive blood pressure meter (softron BP-98AL, Japan) for mice was used for measurement and recording.

### Histopathological Studies

The fixed cardiac tissues were embedded in paraffin and then cut into 5 μm thick sections. H&E staining was used to assess myocardial structural changes. The Masson trichrome staining kit (cat.no. D026-1-3, Nanjing Jiancheng, China) was used to assess the degree of fibrosis. Photomicrographs were taken at ×400 magnification by an intelligent biological microscope (OLYMPUS BX53). The percentage of collagen-stained area (blue) was calculated using a quantitative digital image analysis system (Image-Pro Plus 6.0 software).

### Immunofluorescence

H9C2 cells were fixed with 4% paraformaldehyde for 30 min before permeabilization with 0.1% Triton X-100. Afterward, the cells were incubated with α-actin antibody (1:500, cat.no. sc-32251, Santa Cruz) at 4°C overnight and then with the secondary antibody for 1 h at 37°C. Nuclei were stained with DAPI (cat.no. S2110, Solarbio Science & Technology Co., Ltd., Beijing, China). Images were captured by an inverted fluorescence microscope (OLYMPUS IX51).

### Enzyme-Linked Immunosorbent Assay

The serum levels of the cytokines IL-6 (cat.no. 88-7064-86), TNF-α (cat.no. 88-7324-86), and IL-1β (cat.no. 88-7013-86) were measured using the mouse ELISA kit (eBioscience, San Diego, CA, United States), and concrete experimental steps were performed according to the instructions of the ELISA kit.

### Quantitative Real-Time Polymerase Chain Reaction

Total RNA was isolated from mouse heart tissues and H9C2 cells using TRIzol reagent (Takara, Japan) and reverse transcribed into cDNA with the Takara reverse transcription kit PrimeScript RT Master Mix (cat.no. RR036A). mRNA levels of genes were quantitatively examined using the fluorescence quantitative PCR instrument (Bio-Rad CFX96) with Fast SYBR™ Green Master Mix (cat.no. 4385610, Thermo Fisher, Waltham, MA, United States). For details regarding the primer sequence, refer to supplementary data (Supplementary Table S1). The relative expression of each gene was calculated with the 2^−ΔΔ^Ct method. GAPDH was used as an internal control.

### Western Blot

The heart tissues and H9C2 cells were lysed with RIPA lysis buffer (Beyotime Institute of Biotechnology, Shanghai, China). Protein concentrations were determined using a BCA protein kit (Beyotime, China). The proteins were separated with 8% or 12% sodium dodecyl sulfate polyacrylamide gel electrophoresis (SDS-PAGE) and then transferred to a polyvinylidene fluoride (PVDF) membrane. The PVDF membrane was laid in 5% skim milk for 1 h and then incubated with primary antibodies such as GAPDH (1:10,000, cat.no. 60004-1-Ig, Proteintech), ANP (1:1,000, cat.no. ab180649, Abcam), BNP (1:1,000, cat.no. ab239510, Abcam), COL3A1 (1:1,000, cat.no. 30565S, Cell Signaling Technology), COL1A1 (1:1,000, cat.no. 72026T, Cell Signaling Technology), β-MHC (1:1,000, cat.no. ab-50967, Abcam), JAK2 (1:1,000, cat.no. 3230S, Cell Signaling Technology), p-JAK2 (1:1,000, cat.no. 4406S, Cell Signaling Technology), STAT3 (1:1,000, cat.no. 9139S, Cell Signaling Technology), p-STAT3 (1:1,000, cat.no. 9145S, Cell Signaling Technology), IκBα (1:1,000, cat.no. 4812S, Cell Signaling Technology), p-IκBα (1:1,000, cat.no. 2859T, Cell Signaling Technology), p65 (1:1,000, cat.no. 8242S, Cell Signaling Technology), and p-p65 (1:1,000, cat.no. 3033S, Cell Signaling Technology) at 4°C overnight. After being washed with TBST thrice, the membranes were then incubated with horseradish peroxidase–conjugated goat anti-rabbit or goat anti-mouse secondary antibodies at room temperature for 1 h. The result was detected by an ECL detection kit (Millipore, United States). The dilution of antibodies (cat.no. W019-1-1) was purchased from Nanjing Jiancheng (Nanjing, China). The primary and secondary antibodies were diluted in a ratio of 1:500–10,000 using the dilution of antibodies.

### Determination of Serum Biochemical Indexes

The following biochemical indexes were examined from the experimental mice serum sample: heart function indexes [lactate dehydrogenase (LDH)], liver function indexes [alanine aminotransferase (ALT) and aspartate aminotransferase (AST)], and kidney function indexes [urea formaldehyde (UREA) and creatinine sox (CREA-S)]. All the indexes were detected by the automatic chemistry analyzer (BS-240vet, Mindray Bio-Medical Electronics Co. Ltd., Shenzhen, China).

### Statistical Analysis

All results are presented as mean ± SD and analyzed using GraphPad (version 8.0.1, GraphPad Prism Software). One-way analysis of variance (ANOVA) by Tukey’s multiple-comparisons test was used to compare each variable for differences among the groups. The statistical significance was expressed as *p* < 0.05.

## Results

### Loganin Attenuates Cardiomyocyte Hypertrophy Induced by Ang II *In Vitro*

**FIGURE 1:**
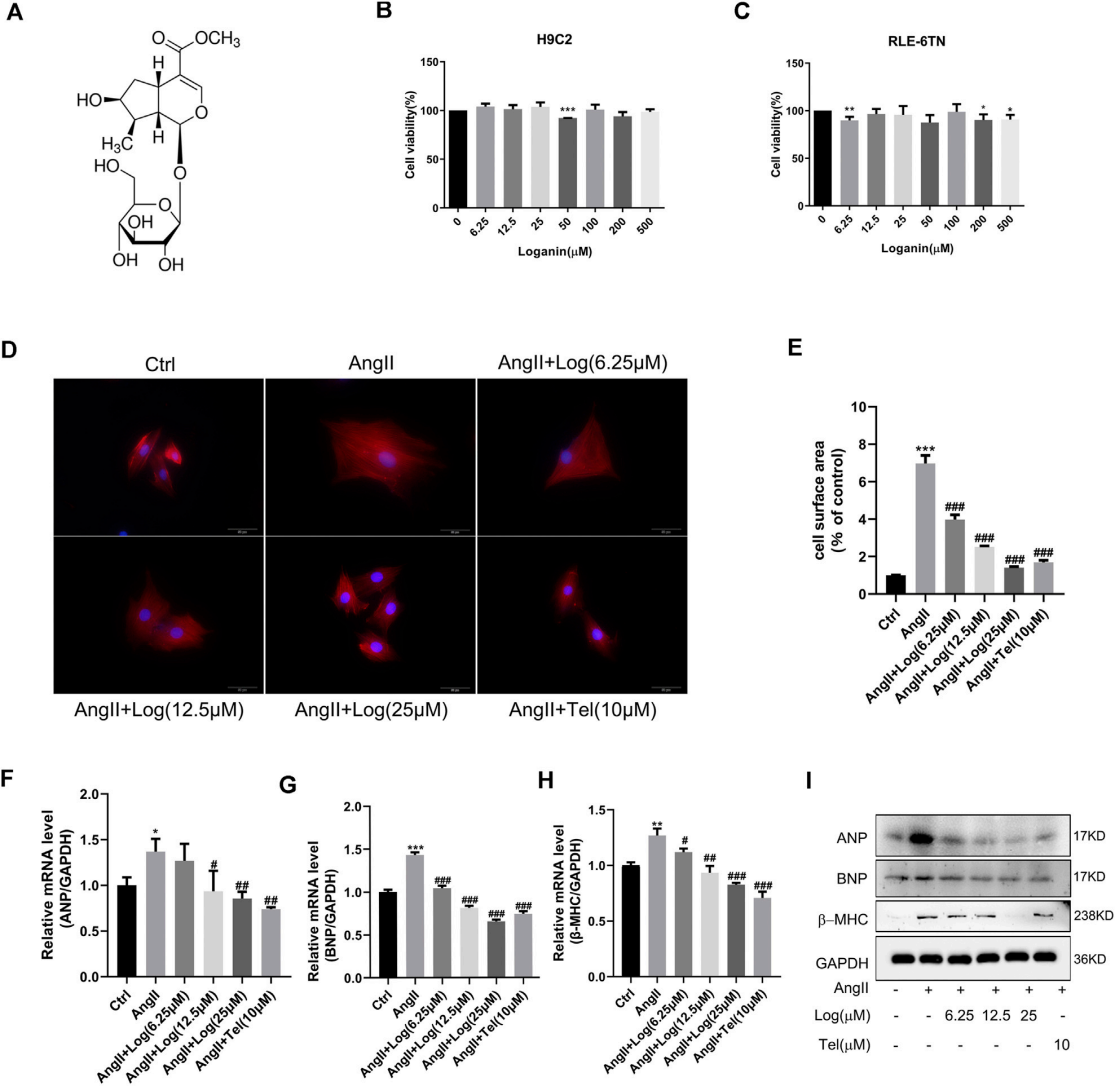
Loganin attenuates cardiomyocyte hypertrophy induced by Ang II *in vitro.* **(A)** Molecular structure of loganin. **(B,C)** H9C2 and RLE-6TN cells are treated with different concentrations of loganin (0–500 μM) for 24 h. The cell viabilities are measured by MTT assay (*n* = 3). **(D)** H9C2 cells are stained with α-actin (red) and DAPI (blue) to detect the size of cells, and immunofluorescence images are captured by fluorescence microscopy. Scale bars = 50 µm. **(E)** Statistical quantitative analysis of the area of H9C2 cells by ImageJ software (*n* = 3). **(F–H)** Loganin suppressed the mRNA expression levels of Ang II–induced hypertrophic markers, namely, ANP, BNP, and β-MHC, using RT-PCR analysis (*n* = 3). **(I)** Relative expression of ANP, BNP, and β-MHC proteins in the Ang II–treated H9C2 cells with or without loganin incubation. Data are expressed as mean ± SD. *p < 0.05, **p < 0.01, ***p < 0.001 vs the control group; #*p* < 0.05, ##*p* < 0.01, ###*p* < 0.001 vs the Ang II–treated group (analyzed using one-way ANOVA). Telmisartan (Tel) is a positive control group.

The molecular structure of loganin is shown in Figure 1A. To investigate the cytotoxic effect of loganin on normal cells, H9C2 and RLE-6TN cell lines were evaluated by MTT assay. As shown in Figures 1B,C, the cell viability was not significantly decreased compared with that in the control group in loganin-treated H9C2 and RLE-6TN cell lines. The MTT assay demonstrates that loganin barely suppresses normal cells. Based on these preliminary data, 6.25, 12.5, and 25 μM were selected in the subsequent experiments.

To the best of our knowledge, Ang II, an important ingredient of the renin–angiotensin system (RAS), plays a key role in stimulating cardiac hypertrophy. Herein, we stimulated cardiomyocyte hypertrophy by incubation with 800 nM Ang II in H9C2 cells. As loganin has not been studied in hypertrophic H9C2 cells, we determined whether loganin possesses cytoprotective effects against cardiomyocyte hypertrophy. At first, we evaluated the size of myocardial cells induced by Ang II with or without loganin and telmisartan treatment *via* immunofluorescence assay. Under a fluorescence microscope, α-actin exhibited bright red fluorescence and the cell nucleus showed blue fluorescence (Figure 1D). In addition, the H9C2 cell surface area was quantified by ImageJ software. As shown in Figure 1E, treatment with Ang II caused enhancement of the cell surface area compared with that in the control group in H9C2 cells, and the surface area of the cells in the loganin-treated group was decreased in a dose-dependent manner compared with that in the Ang II–treated group (telmisartan, as a positive control). Similar morphological changes were obtained by optical microscopy, and the area of the H9C2 cells was calculated (Supplementary Figures S1A,B). In order to further confirm the anti-hypertrophic effect of loganin, we then examined cardiomyocyte hypertrophy markers, namely, ANP (atrial natriuretic peptide), BNP (brain natriuretic peptide), and β-MHC (β-myosin heavy chain), using RT-PCR and western blot analysis in Ang II–induced cells treated with or without loganin. The mRNA expression levels of ANP, BNP, and β-MHC were markedly up-regulated in H9C2 cells treated with Ang II, and loganin significantly decreased the levels upon Ang II stimulation in a concentration-dependent manner (Figures 1F–H). Concomitantly, we determined the protein expression levels of ANP, BNP, and β-MHC in Ang II–treated H9C2 cells. As presented in Figure 1I and Supplementary Figure S3A, loganin dramatically decreased the Ang II–induced protein expression of ANP, BNP, and β-MHC in H9C2 cells. Overall, our results certified that loganin alleviated Ang II–induced cardiomyocyte hypertrophy *in vitro*.

### Loganin Alleviates Hypertension and Cardiac Hypertrophy Induced by Ang II *In Vivo*

**FIGURE 2:**
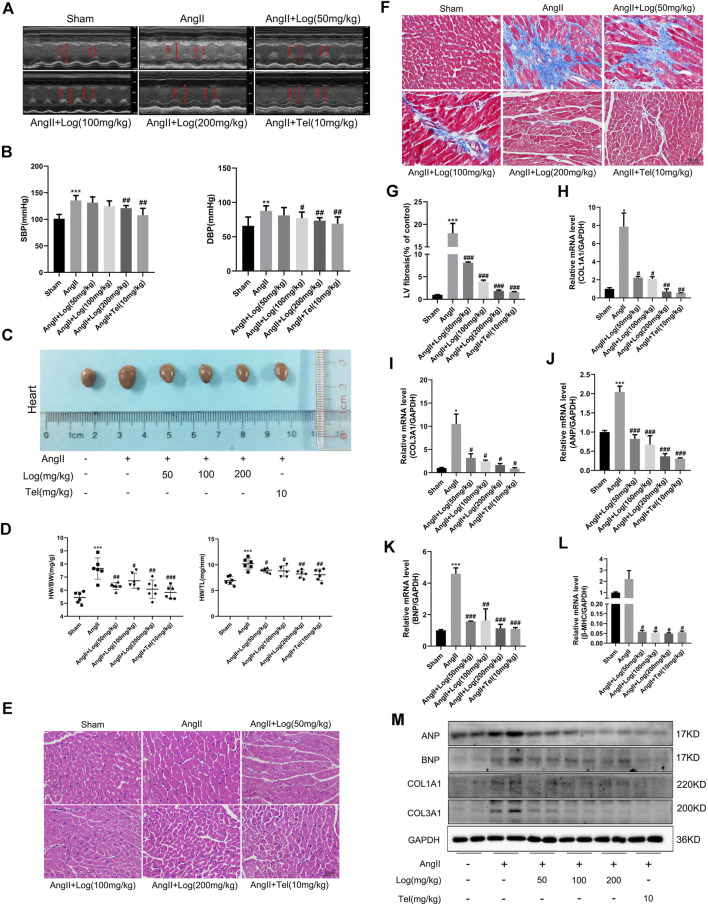
Loganin suppresses cardiac hypertrophy and fibrosis induced by Ang II *in vivo.* **(A)** Representative echocardiography pictures. Echocardiography M-mode imaging obtained from C57BL/6 mice with or/and without Ang II and loganin treatment. M-mode images obtained from short-axis measurements are used to assess cardiac function. Vertical arrows represent the diameters of the left ventricle at end diastolic or systolic stages. **(B)** Statistical results for the systolic blood pressure (SBP) and diastolic blood pressure (DBP). **(C)** Morphological images of mouse hearts are obtained from the sham, Ang II, Ang II + Log (50 mg/kg), Ang II + Log (100 mg/kg), Ang II + Log (200 mg/kg), and Ang II + Tel (10 mg/kg) groups. **(D)** Ratio of heart weight (HW) to body weight (BW) and ratio of HW to tibia length in each group. **(E)** Paraffin sections of cardiac tissues are stained with H&E. Representative images are shown. Scale bar: 50 μm. **(F)** Masson’s trichrome staining of hypertrophic heart is shown. Collagen fibers are stained blue, and the myocardium is stained red. Scale bar: 50 μm. **(G)** Fibrotic areas are quantified in the hearts of mice using ImageJ software. **(H–L)** mRNA expression levels of collagen 1A1 (COL1A1), collagen 3A1 (COL3A1), ANP, BNP, and β-MHC are estimated by RT-PCR and normalized to those of GAPDH. **(M)** Western blot analysis showing ANP, BNP, COL1A1, and COL3A1 protein levels in mouse cardiac tissues. GAPDH is used as a loading control. Data are expressed as mean ± SD (*n* = 3–6). *p < 0.05, **p < 0.01, ***p < 0.001 vs the sham group; #*p* < 0.05, ##*p* < 0.01, ###*p* < 0.001 vs the Ang II–treated group (analyzed using one-way ANOVA). Telmisartan (Tel) is a positive control group.

As far as we know, Ang II acts as a critical effector peptide of RAS that is involved in hypertension, which is a major predisposing reason for the development of cardiac hypertrophy. Thus, we first examined the changes in blood pressure in Ang II–induced C57BL/6 mice treated with or without loganin. As described in Figure 2B, Ang II infusion triggered a hypertensive response, which was obvious elevation of systolic blood pressure (SBP) and diastolic blood pressure (DBP) compared to those in the sham group. Furthermore, mice administered loganin exhibited an evident decrease in blood pressure which was compared to that of the Ang II–treated mice.

In order to further analyze whether loganin alleviates cardiac hypertrophy induced by Ang II in mice, the measurement of cardiac function was performed by echocardiography *in vivo*. As shown in Figure 2A and Table 1, our results exhibited that sustained exposure to Ang II significantly increased the interventricular septum in diastole (IVSd), interventricular septum in systole (IVSs), ejection fraction (EF), fractional shortening (FS), and left ventricular mass (LV mass)/body weight (BW) ratio, when compared with those in the saline-treated sham group. By contrast, treatment with loganin in the Ang II–induced group dramatically decreased the diastolic IVS, systolic IVS, EF, FS, and LV mass/BW ratio to normal levels as compared to those in the Ang II–stimulated group alone. Furthermore, the left ventricular diastolic posterior wall thickness (LVPWd) and left ventricular systolic posterior wall thickness (LVPWs) were remarkably increased in the Ang II–induced group compared to those in the sham group. Notably, a low dose of loganin did not exert apparent reduction in diastolic LVPW and systolic LVPW compared with those in the Ang II–treated group. However, loganin at 200 mg/kg effectively decreased the diastolic LVPW and systolic LVPW. Although the left ventricular diastolic internal diameter (LVIDd) and left ventricular systolic internal diameter (LVIDs) were not significantly different by Ang II stimulation, they tended to decrease. Moreover, the diastolic LVID and systolic LVID in the loganin-treated group were maintained at the same level as in the sham group. The body weight (BW) and heart rate (HR) were not distinctly different in various groups.

**TABLE 1:** Echocardiographic parameters.

Measurements	Sham	Ang II	Ang II + Log (50 mg/kg)	Ang II + Log (100 mg/kg)	Ang II + Log (200 mg/kg)	Ang II + Tel (10 mg/kg)
IVSd (mm)	0.84 ± 0.13	1.22 ± 0.22^**^	0.82 ± 0.04^##^	0.87 ± 0.19^##^	0.82 ± 0.12^##^	0.91 ± 0.13^#^
LVIDd (mm)	3.21 ± 0.36	2.90 ± 0.25	3.13 ± 0.28	3.45 ± 0.40	3.46 ± 0.14	3.69 ± 0.59^##^
LVPWd (mm)	0.75 ± 0.39	1.57 ± 0.40^*^	1.21 ± 0.39	1.33 ± 0.49	0.86 ± 0.22^#^	0.87 ± 0.09^#^
IVSs (mm)	1.17 ± 0.05	1.73 ± 0.12^**^	1.02 ± 0.15^####^	1.20 ± 0.35^###^	1.15 ± 0.20^###^	1.36 ± 0.12^#^
LVIDs (mm)	2.11 ± 0.44	1.70 ± 0.20	2.23 ± 0.46	2.51 ± 0.47	2.21 ± 0.42	2.28 ± 0.74
LVPWs (mm)	1.16 ± 0.20	1.79 ± 0.28^*^	1.44 ± 0.33	1.55 ± 0.42	1.20 ± 0.12^#^	1.26 ± 0.26
LV mass/BW (mg/g)	3.61 ± 0.68	6.53 ± 1.79^***^	4.48 ± 0.56^#^	4.97 ± 0.75	4.30 ± 0.71^##^	4.05 ± 0.77^##^
EF%	70.31 ± 4.93	80.26 ± 3.14^*^	62.96 ± 7.53^####^	63.82 ± 6.39^###^	65.13 ± 2.60^###^	67.51 ± 5.72^##^
FS%	38.59 ± 3.48	48.38 ± 2.97^**^	34.01 ± 4.80^####^	34.8 ± 3.90^####^	34.87 ± 1.68^###^	38.78 ± 7.16^##^
BW (g)	23.92 ± 1.36	23.22 ± 2.75	25.70 ± 1.58	24.68 ± 2.04	24.42 ± 0.88	24.75 ± 2.09
HR (BPM)	426.33 ± 57.70	423.5 ± 28.08	464.17 ± 20.28	461.50 ± 18.71	452.00 ± 56.70	448.33 ± 15.42

Data are presented as mean ± SD (*n* = 6). *p < 0.05, **p < 0.01, ***p < 0.001 vs the sham group; #*p* < 0.05, ##*p* < 0.01, ###*p* < 0.001, ####*p* < 0.0001 vs the Ang II–treated group. Abbreviations: IVSd, interventricular septum in diastole; LVIDd, left ventricular diastolic internal diameter; LVPWd, left ventricular diastolic posterior wall thickness; IVSs, interventricular septum in systole; LVIDs, left ventricular systolic internal diameter; LVPWs, left ventricular systolic posterior wall thickness; LV mass/BW, left ventricular mass/body weight; EF, ejection fraction; FS, fractional shortening; BW, body weight; HR, heart rate.

Next, anatomical parameters were measured in a mouse cardiac hypertrophic model induced by Ang II. First of all, we analyzed the ratio of heart weight (HW) to body weight (BW) and the ratio of HW to tibia length (TL) as indexes of cardiac hypertrophy. As depicted in Figure 2D, loganin treatment in Ang II–induced cardiac hypertrophic mice results in a significant reduction in HW/BW ratio (mg/g) and HW/TL ratio (mg/mm). In addition, we assessed the macroscopic difference in hearts stimulated by Ang II in the absence or presence of loganin treatment. The morphological changes are shown in Figure 2C; the size of hearts was obviously elevated when induced by Ang II, and the heart size was evidently smaller in the loganin-treated group. H&E staining of histological sections further demonstrated the inhibitory effect of loganin on cardiac hypertrophy induced by Ang II in the hearts of C57BL/6 mice (Figure 2E). Subsequently, we examined the left ventricular mRNA expression of fetal genes, ANP, BNP, and β-MHC, associated with cardiac hypertrophy. RT-PCR results showed that Ang II infusion induced a significant enhancement in the mRNA levels of these genes, which was strikingly suppressed by loganin treatment (Figures 2J–L). Besides, similar results were obtained when we detected the protein expression levels of ANP and BNP by western blot analysis. As expected, the protein expressions of hypertrophic markers ANP and BNP were apparently elevated in the Ang II–stimulated group when compared with the sham group, whereas these elevated expressions of ANP and BNP in the Ang II–stimulated group were again remarkably reduced in the loganin-treated group (Figure 2M and Supplementary Figure S3B).

On the basis of these experimental results, we suggested that loganin protects against hypertension and cardiac hypertrophy induced by Ang II *in vivo*. Interestingly, the improvements of cardiac function and left ventricular structure were comparable between loganin and telmisartan, which was applied in patients with hypertension and cardiac hypertrophy.

### Loganin Ameliorates Ang II–Induced Cardiac Fibrosis and Inflammation

Pathological cardiac hypertrophy is a pivotal risk factor in the development of heart failure and usually causes cardiac fibrosis and inflammation. At first, to evaluate the effect of loganin on Ang II–caused cardiac fibrosis, paraffin-embedded slides were stained with Masson’s trichrome staining kit. Under normal circumstances, collagen fibers were stained blue and the myocardium was stained red. As shown in Figures 2F,G, striking cardiac fibrosis was observed in the Ang II–treated mice. In the loganin-treated mice, the extent of cardiac fibrosis was effectively decreased in a concentration-dependent manner compared with that in the Ang II–treated mice. Furthermore, we examined the expression of fibrosis marker genes. As depicted in Figures 2H,I, the mRNA expression levels of cardiac fibrosis markers, collagen 1A1 and collagen 3A1, were greatly elevated in the Ang II–treated group when compared to the sham saline-treated group, but loganin remarkably inhibited the elevation of COL1A1 and COL3A1 mRNA levels when compared with those in the Ang II–treated group. Likewise, western blots validated that protein levels of COL1A1 and COL3A1 apparently decreased in the loganin-treated group as compared to the Ang II–treated group alone (Figure 2M and Supplementary Figure S3B).

**FIGURE 3:**
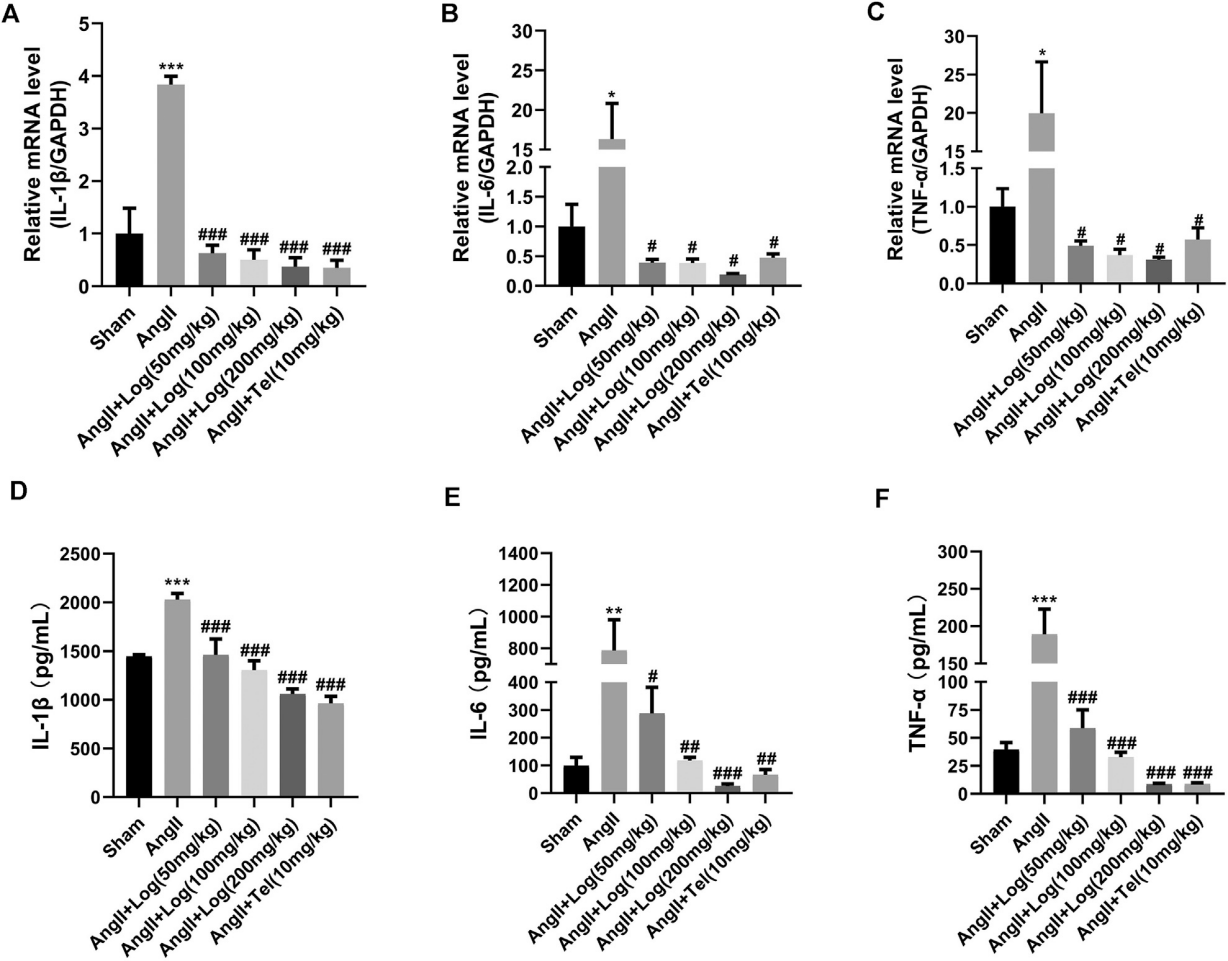
Loganin counteracts inflammation stimulated by Ang II in mice. **(A–C)** The mRNA expression of IL-1β, IL-6, and TNF-α is examined by RT-PCR (*n* = 3). **(D–F)** Circulating IL-1β, IL-6, and TNF-α serum levels. These pro-inflammatory factors are tested by ELISA (*n* = 3). Data are expressed as mean ± SD. *p < 0.05, **p < 0.01, ***p < 0.001 vs the sham group; #*p* < 0.05, ##*p* < 0.01, ###*p* < 0.001 vs the Ang II–treated group. Telmisartan (Tel) is a positive control group.

Elevated pro-inflammatory cytokine secretion in cardiac tissue is a critical characteristic of cardiac hypertrophy induced by Ang II (Forrester et al., 2018). Therefore, in order to explore the anti-inflammatory effects of loganin on cardiac hypertrophy caused by Ang II, RT-PCR analysis was performed *in vivo*. As shown in Figures 3A–C, loganin treatment remarkably blocked the mRNA expression of IL-1β, IL-6, and TNF-α compared to those in the Ang II–treated group. Moreover, ELISA results showed that treatment of Ang II clearly augmented circulating pro-inflammatory factors such as IL-1β, IL-6, and TNF-α serum levels, whereas loganin could effectively suppress the pro-inflammatory factor expression *in vivo* (Figures 3D–F; Supplementary Figures S4). Similar to the *in vivo* results, the mRNA expression levels of IL-1β, IL-6, and TNF-α were markedly reduced using RT-PCR analysis in H9C2 cells treated with loganin when compared with the Ang II treatment alone (Supplementary Figures S2A–C).

Collectively, these results revealed that Ang II stimulation dramatically increased cardiac fibrosis and inflammation, which were largely blunted by loganin treatment.

### Loganin Inhibits Ang II–Stimulated Activation of JAK2/STAT3 and NF-κB Signaling Pathways

**FIGURE 4:**
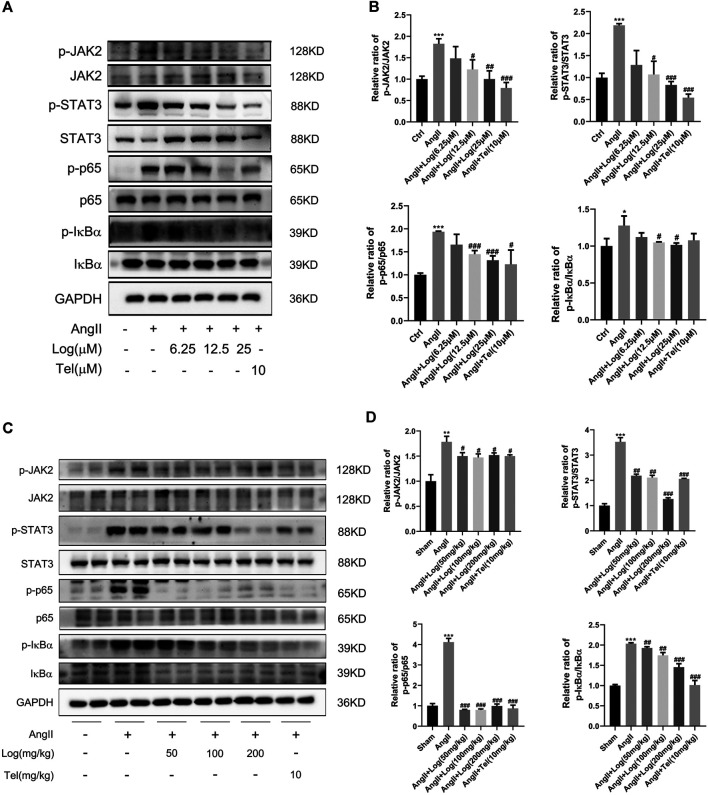
Loganin inhibits Ang II–induced activation of the JAK2/STAT3 and NF-κB signaling pathways. **(A,C)** The expression levels of signaling pathway proteins (total and phosphorylation of JAK2, STAT3, p65, and IκBα) are determined by western blot *in vitro* and *in vivo*. **(B,D)** Quantification of the relative changes in phosphorylation of JAK2, STAT3, p65, and IκBα. GAPDH is used for normalization. Data are expressed as mean ± SD (*n* = 3). *p < 0.05, **p < 0.01, ***p < 0.001 vs the control/sham group; #*p* < 0.05, ##*p* < 0.01, ###*p* < 0.001 vs the Ang II–treated group (analyzed using one-way ANOVA). Telmisartan (Tel) is a positive control group.

To investigate the mechanisms of loganin acting on cardiac hypertrophy, we detected effects of loganin on the JAK2/STAT3 and NF-κB signaling pathways, which are involved in cardiac hypertrophy and inflammation induced by Ang II. Among the signaling pathway proteins, we found that the protein expression levels of phosphorylated JAK2, phosphorylated STAT3, phosphorylated p65, and phosphorylated IκBα induced by Ang II were markedly increased in H9C2 cells. However, this expression was apparently reduced following loganin treatment (Figure 4A). Consistent with the results *in vitro*, the phosphorylation of JAK2, STAT3, p65, and IκBα stimulated by Ang II was obviously decreased *in vivo* when treated with loganin (Figure 4C). Meanwhile, statistical results showed that the ratios of p-JAK2/JAK2, p-STAT3/STAT3, p-p65/p65, and p-IκBα/IκBα were dramatically reduced after treatment with various concentrations of loganin *in vivo* and *in vitro* (Figures 4B,D). As a whole, these findings indicated that loganin could inhibit Ang II–induced activation of JAK2/STAT3 and NF-κB signaling pathways by suppressing the phosphorylation of critical proteins.

### Loganin Has No Obvious Toxicity or Side Effects on Normal Organs

**FIGURE 5:**
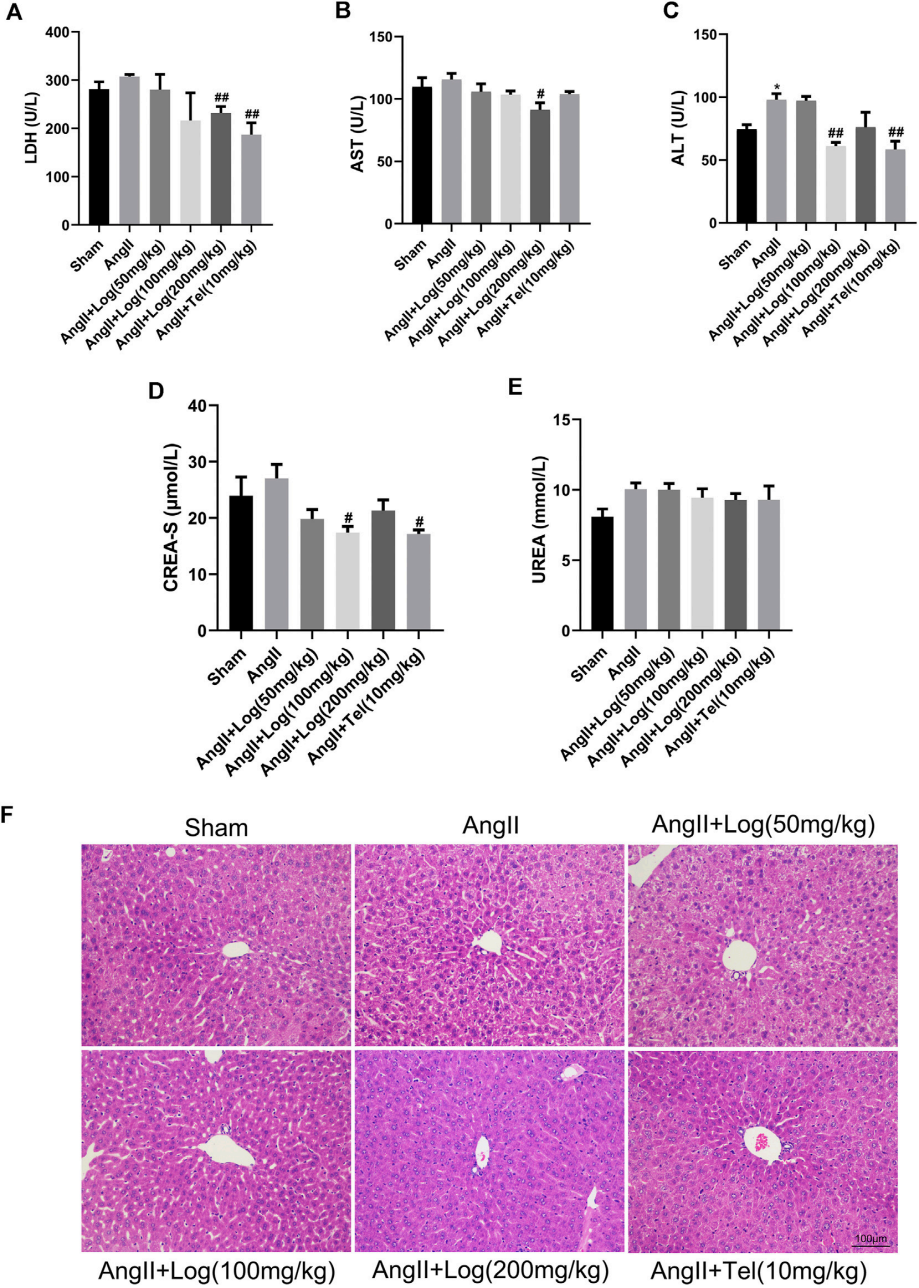
Loganin has no obvious toxicity or side effects on normal organs. **(A–E)** The levels of LDH, AST, ALT, CREA-S, and UREA are tested from the serum of various mice groups (*n* = 3). **(F)** Liver histopathology is done by H&E staining. Scale bar = 100 µm. Data are expressed as mean ± SD. *p < 0.05 vs the sham group; #*p* < 0.05, ##*p* < 0.01 vs the Ang II–treated group (analyzed using one-way ANOVA).

Unexpected adverse effects are major obstacles of clinical applications of common therapeutic drugs, such as cardiotoxicity, hepatotoxicity, and nephrotoxicity. Consequently, we detected the possible toxicity or adverse effects of loganin. The biochemical indexes, including LDH, AST, ALT, CREA-S, and UREA, were measured with the blood serum of the various mice groups. As presented in Figures 5A–E, the levels of these biochemical indexes were elevated in the Ang II–exposed group compared with sham but were ameliorated or obviously diminished upon loganin treatment. These results showed that loganin had no damage to the heart, liver, and kidney and even had protective effect. Furthermore, the conclusion was supported by similar data from H&E staining analysis as no obvious hepatomegaly and significantly enhanced necrotic hepatocytes were observed (Figure 5F). Considering these findings, we suggested that loganin has no obvious toxicity or side effects on normal organs.

## Discussion

**FIGURE 6:**
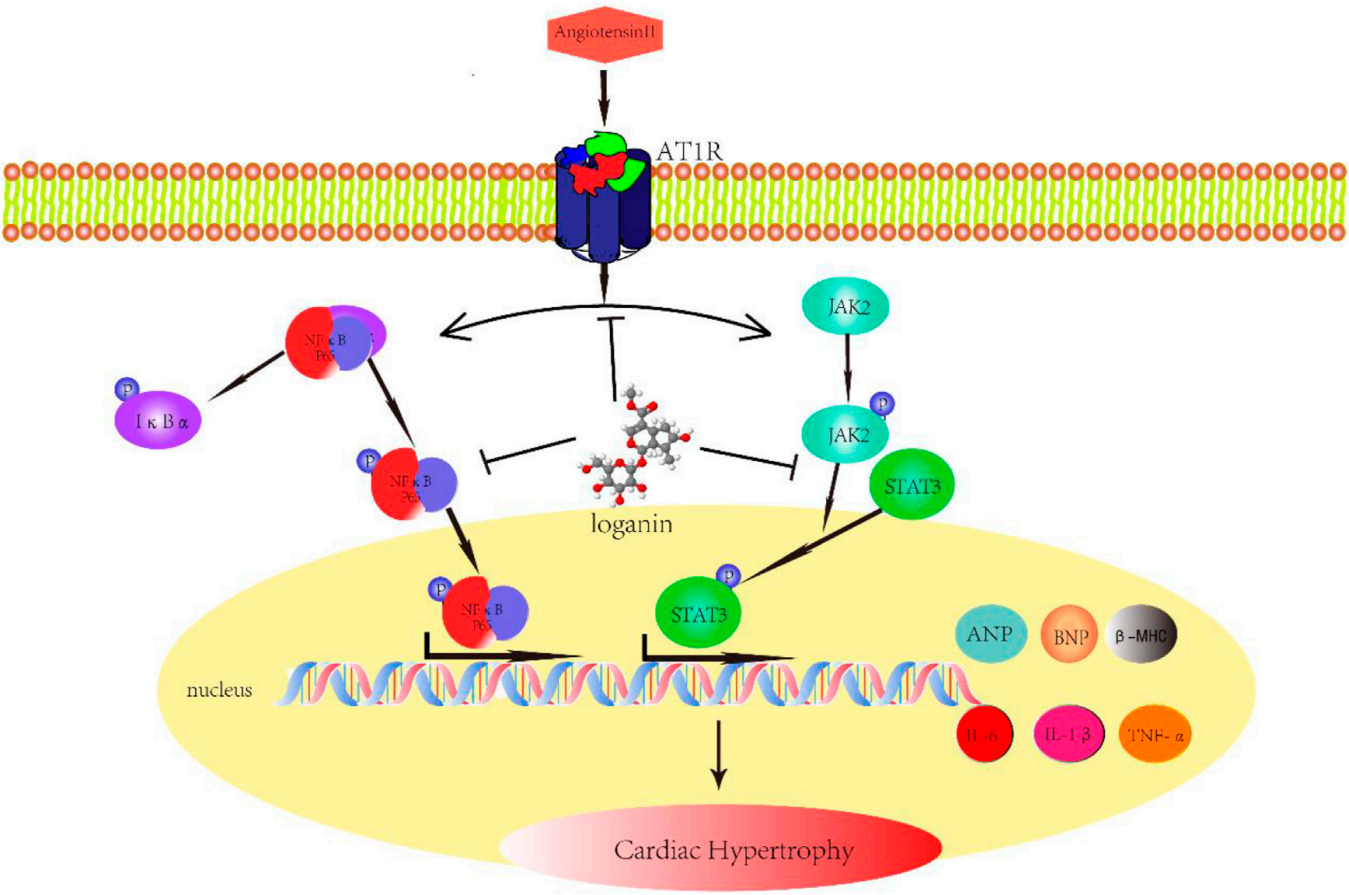
Schematic diagram with roles of loganin in Ang II–induced cardiac hypertrophy. Angiotensin II (Ang II) can induce cardiac hypertrophy *in vivo* and *in vitro*, which is mainly mediated through angiotensin II type 1 receptor (AT1R). Given Ang II stimulation, in addition to the increase in IL-1β, IL-6, and TNF-α secretion, ANP, BNP, and β-MHC expressions are also augmented. Loganin ameliorates Ang II–provoked cardiac hypertrophy and cardiac damage through suppressing the JAK2/STAT3 and NF-κB signaling pathways.

In this study, we reported for the first time that loganin inhibits Ang II–induced cardiac hypertrophy and cardiac damage *in vivo* and *in vitro.* Furthermore, our results suggested that loganin markedly ameliorates cardiac fibrosis and inflammation induced by Ang II. Our mechanism data revealed that the JAK2/STAT3 and NF-κB signaling pathways are closely associated with the cardioprotective effect of loganin (Figure 6). Besides, the outstanding findings of the present study were to prove that loganin has no distinct toxicity or side effects on normal organs. Collectively, our research indicated that loganin may be a promising candidate drug for the treatment of cardiac hypertrophy and heart failure.

Under pathological circumstances, cardiac hypertrophy is a critical risk factor in the progression of heart failure and usually results in cardiac injury and cardiac dysfunction (Jiang et al., 2018). Currently, although the pathophysiological mechanisms of cardiac hypertrophy have been investigated in many reports, the clinical therapeutic effect for patients with cardiac hypertrophy is still limited. Consequently, novel effective drugs need to be studied for the prevention and treatment of cardiac hypertrophy. In recent years, evidence from numerous studies indicated that natural products extracted from traditional Chinese medicine are shown to have cardioprotective effect and are generally used in therapy of cardiovascular diseases (Wu et al., 2017; Bian et al., 2020). As a monomer compound, loganin has been found in many medicinal plants, including *Cornus officinalis*, Flos Lonicerae, and *Strychnos nux*-*vomica* (Frederich et al., 2004; Li et al., 2016)*.* There is increasing evidence that loganin exerted huge beneficial effects in different disease models. For instance, loganin could improve long-term learning-and-memory–deficit disorders induced by scopolamine and may have remarkable therapeutic value for the therapy of Alzheimer’s disease (Hwang et al., 2017). Similarly, Shirin and colleagues revealed that acute administration of loganin could enhance spatial memory in diabetic rats (Babri et al., 2013). Moreover, Wen *et al*. (2020) illustrated that loganin could ameliorate burn injuries and significantly reduce the production of inflammatory cytokines and oxidative stress by repressing the activation of the TLR4/NF-κB signaling pathway. To the best of our knowledge, only one study has implied that loganin appears to suppress the progression of atherosclerosis (Li et al., 2016). Thus, it is not clear whether loganin has therapeutic effect on cardiovascular diseases. In our experiments, we investigated the relationship between loganin and cardiac hypertrophy based on cell and animal models. Traditionally, telmisartan is one of the Ang II receptor blockers (ARBs), and it has been used to treat hypertension in the clinic. It is well-documented that hypertension generally enhances ventricular afterload and thereby promotes cardiac hypertrophy. A study of Li *et al*. (2017) manifested that telmisartan can effectively suppress cardiac hypertrophy and cardiomyocyte apoptosis through inhibiting the NFAT/ANP/BNP signaling pathways. Thence, we assessed the therapeutic effects of loganin on this model of cardiac hypertrophy, using telmisartan as a positive control. We found that loganin can inhibit Ang II–induced cardiac hypertrophy *in vitro* and *in vivo*, respectively. The therapeutic value of high-dose loganin was comparable to that of treatment with telmisartan.

Cardiac fibrosis is an important phenotype of pathological cardiac hypertrophy and usually characterized by enhanced levels of collagen (Nakamura and Sadoshima, 2018). In the current study, we found that loganin notably decreased cardiac fibrosis induced by Ang II. Further mRNA and protein detection revealed that loganin effectively reduced the expression of collagen 1A1 and collagen 3A1 *in vivo*. These findings suggested that loganin significantly ameliorated Ang II–induced cardiac fibrosis in mice.

The pro-inflammatory cytokines are small molecular proteins, which have been reported to exert detrimental effects on the heart and are involved in pathological cardiac hypertrophy (Oldfield et al., 2020). Over the past two decades, many studied have indicated that IL-1β, IL-6, and TNF-α are closely implicated in cardiac fibrosis, pathological cardiac remodeling, and cardiac hypertrophy (Sun et al., 2007; Coles et al., 2007; Cannon, 2000; Melendez et al., 2010). In the present study, we examined expression levels of the pro-inflammatory cytokines IL-1β, IL-6, and TNF-α in H9c2 cells and in mice. Our data certified that the levels of these cytokines were evidently reduced when treated with loganin compared with the Ang II incubation alone. Furthermore, Ang II employs the JAK2/STAT3 and NF-κB signaling pathways in mediating inflammatory response and cardiac hypertrophy (Mei et al., 2020; Han et al., 2017). According to published studies, TNF-α plays a critical role in triggering the activation of the NF-κB signaling pathway, and this activation was mediated by TNF-α receptors (Rohini et al., 2010). In addition, IL-6 activates the JAK2/STAT3 signaling pathway in cardiac hypertrophy, and it is produced by macrophages and cardiomyocytes in response to hypertrophic stimulus (Tham et al., 2015). Chen *et al*. (2020a) showed that loganin evidently alleviated diabetes mellitus (DM)–induced reproductive damage partially by regulating the NF-κB signaling pathway. Chu *et al*. (2020) reported that loganin can protect against chronic constriction injury–induced neuroinflammation and pain behavior by suppressing TNF-α/IL-1β–dependent NF-κB activation. Chen *et al*. (2020b) found that loganin effectively inhibited the apoptosis of podocytes upon diabetic nephropathy by downregulating the RAGE/p38 MAPK/NF-κB pathway. In our study, we have clearly proven that loganin dramatically reduced the expression of p-JAK2, p-STAT3, p-p65, and p-IκBα compared to those in the Ang II–treated group. Based on these results, we concluded that loganin mitigated Ang II–provoked cardiac hypertrophy at least partially through inhibiting the JAK2/STAT3 and NF-κB signaling pathways. Nevertheless, more rigorous and detailed studies are required to detect the precise molecular pathways for anti-hypertrophic effects of loganin in the future.

As we mentioned before, several studies have indicated that loganin possesses protective properties on some diseases. For example, a previous report inferred that loganin has protective effects on MPTP-triggered Parkinson’s disease (PD) mice *via* suppressing autophagy, inflammatory responses, and the loss of dopaminergic neurons (Xu et al., 2017). Analogously, a study by Kwon *et al*. (2011) implied that loganin exerts neuroprotective effects by inhibiting hydrogen peroxide–induced apoptosis in neuronal cells and that this compound might be developed as a clinical drug to alleviate neurodegenerative diseases. In this regard, we systematically investigated the possible toxic or undesirable side effects of loganin in our experiments. The results demonstrated that loganin did not cause obvious toxicity or adverse effects on normal organs such as the heart, liver, and kidney.

In summary, the present study illustrated for the first time that loganin distinctly suppressed Ang II–mediated cardiac hypertrophy in H9C2 cells and in mice. Moreover, loganin can achieve cardioprotective effects through attenuating cardiac fibrosis, decreasing pro-inflammatory cytokine secretion, and suppressing the activation of JAK2/STAT3 and NF-κB signaling pathways. Finally, we demonstrate that loganin has no significant toxicity or side effects on normal cells and organs. Accordingly, the natural product, loganin, might be a novel effective agent for the treatment of cardiac hypertrophy and heart failure.

## Data Availability

The raw data supporting the conclusions of this article will be made available by the authors, without undue reservation.

## Supplementary Material

Supplementary Figure 1 The area of H9C2 cells is detected by light microscopy. **(A)** H9C2 cells are treated with various concentrations of Log (0, 6.25, 12.5, and 25 μM) for 24 h in the presence of 800 nM Ang II. Representative images of H9C2 cells by light microscopy. Scale bars = 100 µm. **(B)** H9C2 cell area quantification by ImageJ software. All values are expressed as mean ± SD (*n* = 6). ****p* < 0.001 vs the control group; #*p* < 0.05, ##*p* < 0.01 vs the Ang II–treated group. Tel is a positive group.

Supplementary Figure 2 Loganin blocks the mRNA expression of pro-inflammatory cytokines in H9C2 cells. **(A–C)** The mRNA expression of IL-1β, IL-6, and TNF-α is examined by RT-PCR. Data are expressed as mean ± SD (*n* = 3). **p* < 0.05, ***p* < 0.01, ****p* < 0.001 vs the control group; #*p* < 0.05, ##*p* < 0.01, ###*p* < 0.001 vs the Ang II–treated group. Tel is a positive group.

Supplementary Figure 3 Densitometric analysis is used to evaluate the relative ratios of each protein. GAPDH is used for normalization. Data are expressed as mean ± SD (*n* = 3). **p* < 0.05, ***p* < 0.01, ****p* < 0.001, *****p* < 0.0001 vs the control/sham group; #*p* < 0.05, ##*p* < 0.01, ###*p* < 0.001, ####*p* < 0.0001 vs the Ang II–treated group (analyzed using one-way ANOVA). Telmisartan (Tel) is a positive control group.

Supplementary Figure 4 Standard curve of mouse IL-1β, IL-6, and TNF-α. These pro-inflammatory factors are tested by the ELISA kit.
