# Supplementary figures and images for: Loganin Inhibits Angiotensin II–Induced Cardiac Hypertrophy Through the JAK2/STAT3 and NF-κB Signaling Pathways
Source: Front Pharmacol. 2021 Jun 14;12:678886. doi: 10.3389/fphar.2021.678886 (PMC8237232; doi:10.3389/fphar.2021.678886)

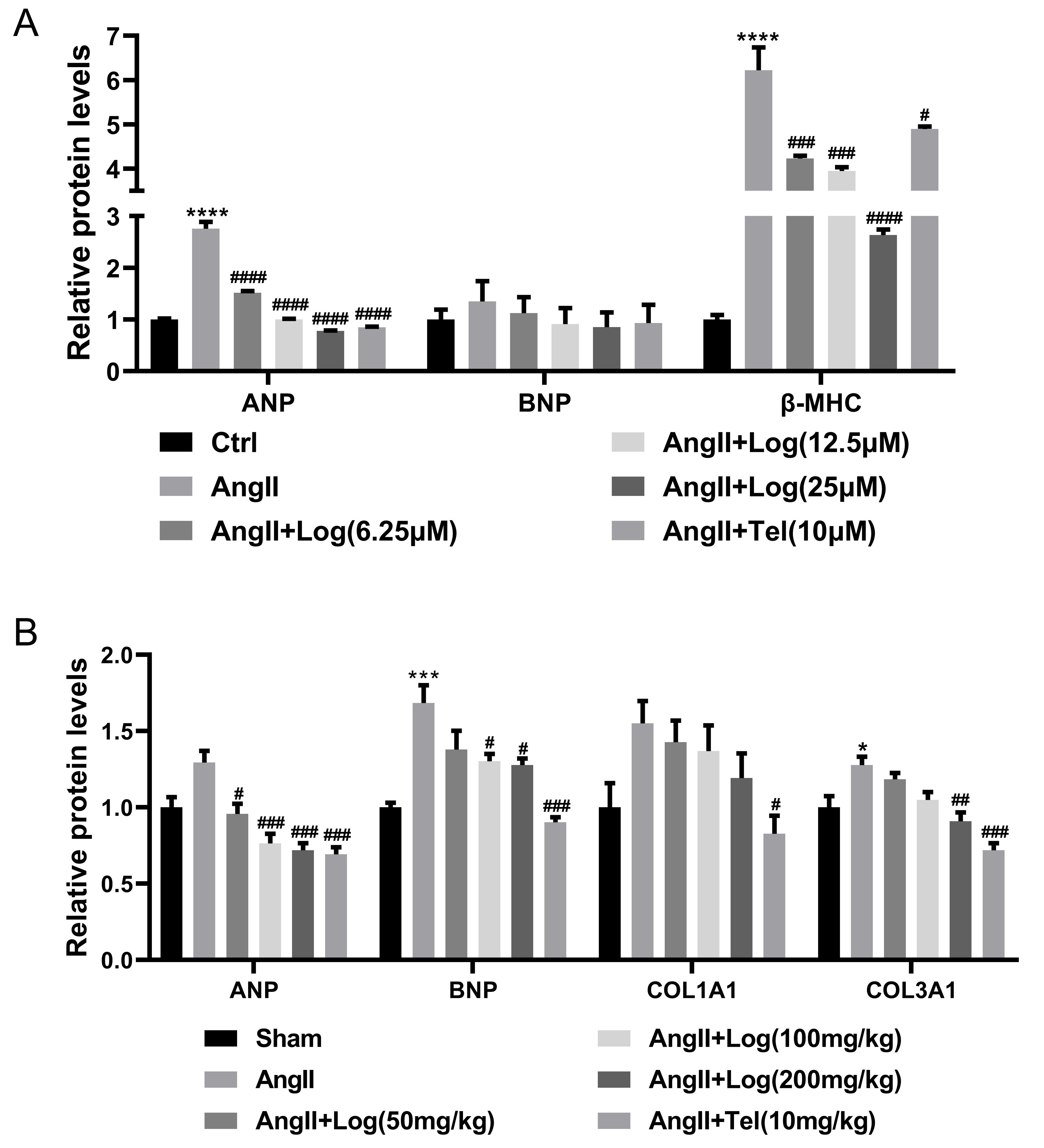

Supplement: Supplementary file 1 [file Image3.JPEG]

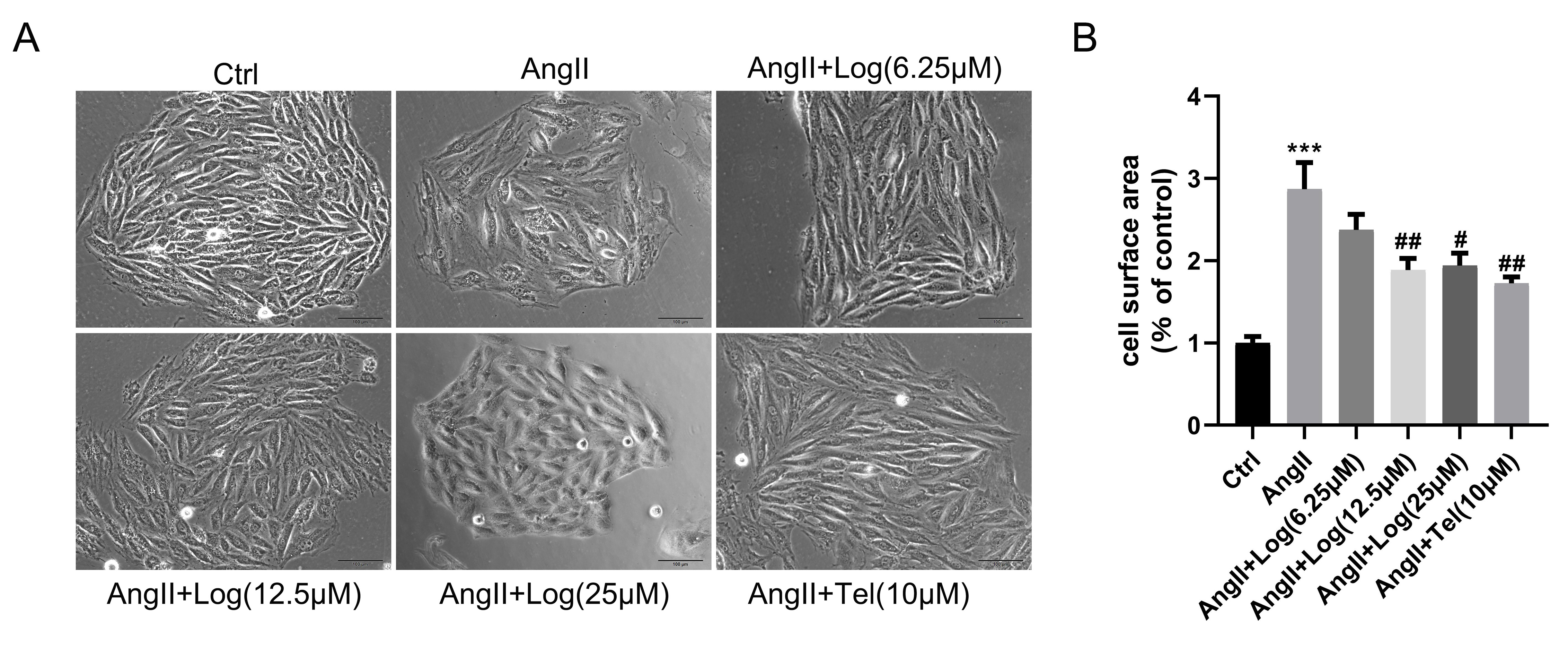

Supplement: Supplementary file 3 [file Image1.JPEG]

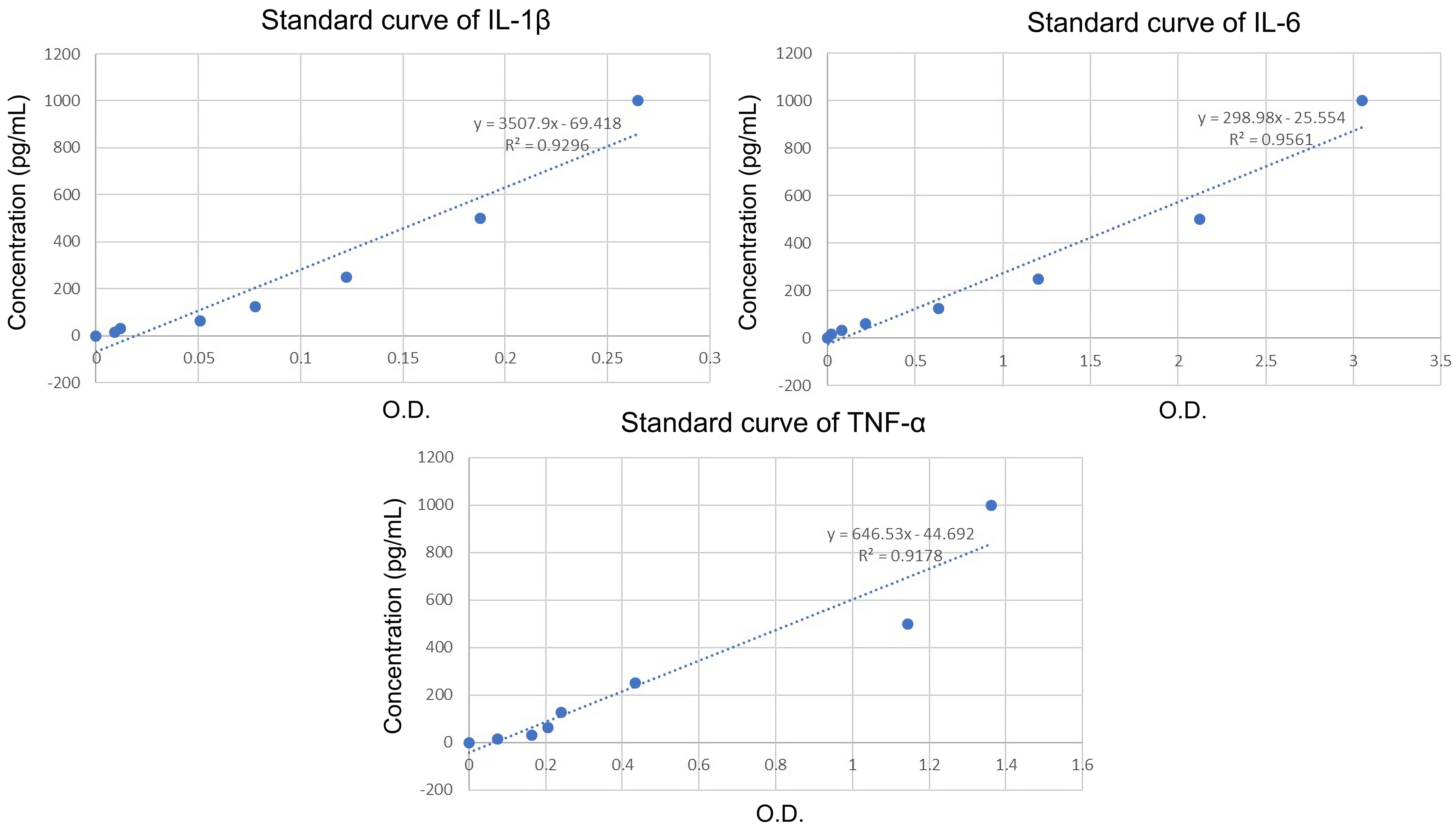

Supplement: Supplementary file 4 [file Image4.JPEG]

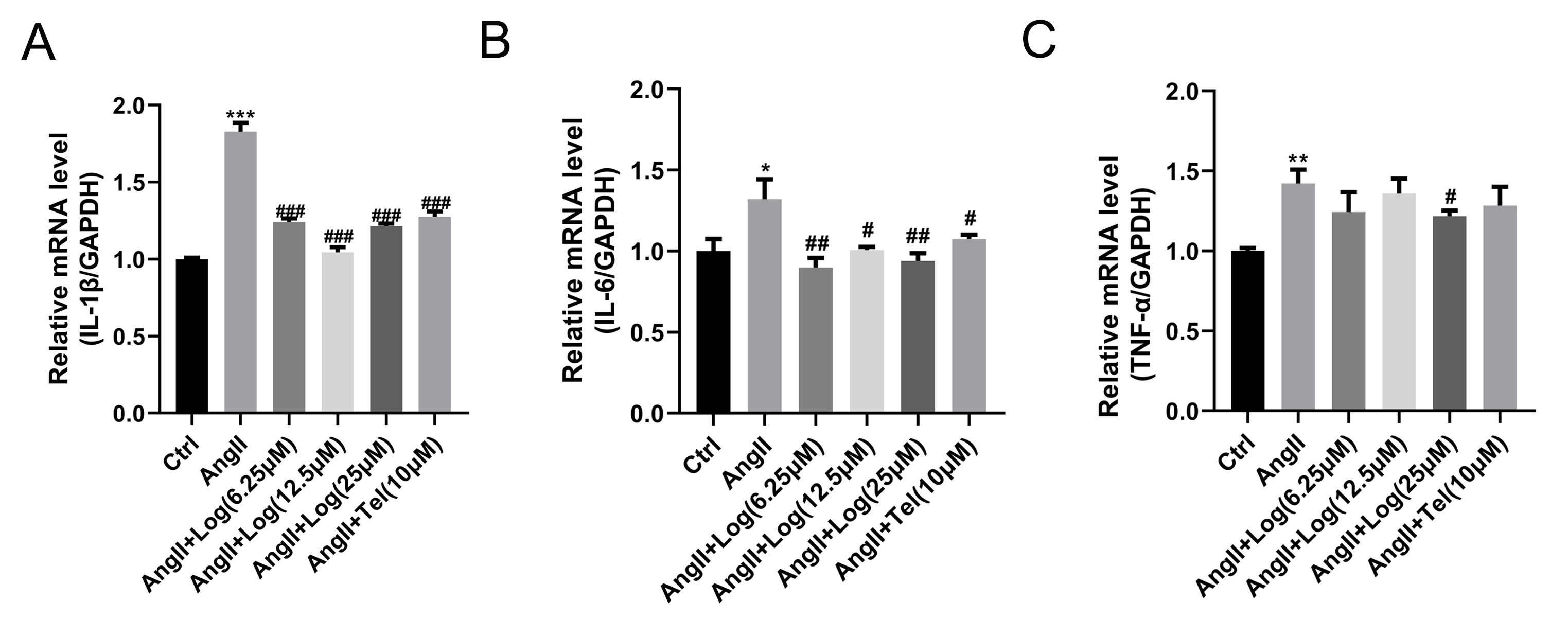

Supplement: Supplementary file 5 [file Image2.JPEG]
