# Supplementary material for: Loganin Inhibits Angiotensin II–Induced Cardiac Hypertrophy Through the JAK2/STAT3 and NF-κB Signaling Pathways
Source: Front Pharmacol. 2021 Jun 14;12:678886. doi: 10.3389/fphar.2021.678886 (PMC8237232; doi:10.3389/fphar.2021.678886)
Supplement: Supplementary file 2 [file Table1.DOCX]

Supplementary table.1. List of primers

| Target gene Sequences Target gene Sequences | | | | | |
| --- | --- | --- | --- | --- | --- |
| Mouse GAPDH | Forward | 5’-GGTTGTCTCCTGCGACTTCA-3’ | Rat GAPDH | Forward | 5’-GGCAAGTTCAATGGCACAGT-3’ |
|  | Reverse | 5’-GGTGGTCCAGGGTTTCTTACTC-3’ |  | Reverse | 5’-TGGTGAAGACGCCAGTAGACTC-3’ |
| Mouse ANP | Forward | 5’-CACAGATCTGATGGATTTCAAGA-3’ | Rat ANP | Forward | 5’-CTTCTCCATCACCAAGGGCTT-3’ |
|  | Reverse | 5’-CCTCATCTTCTACCGGCATC-3’ |  | Reverse | 5’-GGATTTGCTCCAATATGGCCT-3’ |
| Mouse BNP | Forward | 5’-GAAGGTGCTGTCCCAGATGA-3’ | Rat BNP | Forward | 5’-TGATTCTGCTCCTGCTTTTC-3’ |
|  | Reverse | 5’-CCAGCAGCTGCATCTTGAAT-3’ |  | Reverse | 5’-GTGGATTGTTCTGGAGACTG-3’ |
| Mouse IL-1β | Forward | 5’-TCGCAGCAGCACATCAACAAGAG-3’ | Rat IL-1β | Forward | 5’-CTCACAGCAGCATCTCGACAAGAG-3’ |
|  | Reverse | 5’-AGGTCCACGGGAAAGACACAGG-3’ |  | Reverse | 5’-TCCACGGGCAAGACATAGGTAGC-3’ |
| Mouse TNF-a | Forward | 5’-GCGACGTGGAACTGGCAGAAG-3’ | Rat TNF-a | Forward | 5’-TTGGGTTATGCCAAAGATGTTG-3’ |
|  | Reverse | 5’-GCCACAAGCAGGAATGAGAAGAGG-3’ |  | Reverse | 5’-GCTGTGTACGGCTTATTTTCAA-3’ |
| Mouse IL-6 | Forward | 5’-CTTCTTGGGACTGATGCTGGTGAC-3’ | Rat IL-6 | Forward | 5’-ATGATGAGAAACGAGCCAATTG-3’ |
|  | Reverse | 5’-AGGTCTGTTGGGAGTGGTATCCTC-3’ |  | Reverse | 5’-GCTTTGGCTTCTTTCTTACGAG-3’ |
| Mouse β-MHC | Forward | 5’-AGCTCCTGGAAAGAAACACTAA-3’ | Rat β-MHC | Forward | 5’-TTCGGGCGAGTCAAAGATGC-3’ |
|  | Reverse | 5’-GTAACAGTACTTGGCATACGTG-3’ |  | Reverse | 5’-CCTTGTTCTCTGTTGCGTGC-3’ |
| Mouse COL1A1 | Forward | 5’-GCTCCTCTTAGGGGCCACT-3’ |  |  |  |
|  | Reverse | 5’-CCACGTCTCACCATTGGGG-3’ |  |  |  |
| Mouse COL3A1 | Forward | 5’-CTGTAACATGGAAACTGGGGAAA-3’ |  |  |  |
|  | Reverse | 5’-CCATAGCTGAACTGAAAACCACC-3’ |  |  |  |
|  |  |  |  |  |  |
